# Bringing a socially assistive robot to the paediatric emergency department: design, development, and usability testing

**DOI:** 10.3389/frobt.2025.1614444

**Published:** 2025-10-30

**Authors:** Mary Ellen Foster, Jennifer N. Stinson, Lauren Harris, Kate Kyuri Kim, Sasha Litwin, Patricia Candelaria, Summer Hudson, Julie Leung, Ronald P. A. Petrick, Alan Lindsay, Andrés Ramírez-Duque, David Harris Smith, Frauke Zeller, Samina Ali

**Affiliations:** 1 School of Computing Science, University of Glasgow, Glasgow, United Kingdom; 2 Research Institute, The Hospital for Sick Children, Toronto, ON, Canada; 3 Department of Pediatrics, Faculty of Medicine, University of Toronto, Toronto, ON, Canada; 4 Department of Pediatrics, Faculty of Medicine & Dentistry, University of Alberta, Edmonton, AB, Canada; 5 Department of Computer Science, Heriot-Watt University, Edinburgh, United Kingdom; 6 Department of Communication Studies & Media Arts, McMaster University, Hamilton, ON, Canada; 7 Institute for Design Informatics, University of Edinburgh, Edinburgh, United Kingdom; 8 Women and Children’s Health Research Institute, Faculty of Medicine & Dentistry, University of Alberta, Edmonton, AB, Canada

**Keywords:** socially assistive robots, participatory design, real-world evaluation, system validation, technology adoption

## Abstract

**Introduction:**

Children undergoing medical procedures in paediatric Emergency Departments (EDs) often experience significant pain and distress. Socially Assistive Robots (SARs) offer a promising avenue for delivering distraction and emotional support in these high-pressure environments. This study presents the design, development, and formative evaluation of an AI-enhanced SAR to support children during intravenous insertion (IVI) procedures.

**Methods:**

The robot system was developed through a participatory design process involving healthcare professionals, patients, caregivers, and interdisciplinary research teams. The SAR was designed to autonomously adapt its behaviour to the child’s affective state using AI planning and social signal processing. A two-cycle usability study was conducted across two Canadian paediatric EDs, involving 25 children and their caregivers. Feedback was collected through observations, interviews, and system logs.

**Results:**

The SAR was successfully integrated into clinical workflows, with positive responses from children, caregivers, and healthcare providers. Usability testing identified key technical and interaction challenges, which were addressed through iterative refinement. The final system demonstrated robust performance and was deemed ready for a formal randomised controlled trial.

**Discussion:**

This work highlights the importance of co-design, operator control, and environmental adaptability in deploying SARs in clinical settings. Lessons learned from the development and deployment process informed six concrete design guidelines for future SAR implementations in healthcare.

## 1 Introduction

In clinical settings, children regularly experience pain and distress, which can produce adverse effects in both the short term (e.g., fear, agitation, and inability to complete medical procedures) and the long term (e.g., needle phobia, anxiety, heathcare avoidance) (Stevens et al., 2011). While a range of techniques have been shown to help reduce distress (e.g., breathing exercises, distraction techniques, cognitive-behavioural strategies (Chambers et al., 2009)), delivered through a variety of means (e.g., distraction cards, kaleidoscopes, music, virtual reality games), more recent studies have demonstrated that Socially Assistive Robots (SARs) can also be effectively used to manage child pain and distress during medical procedures (Pan et al., 2024). The Emergency Department (ED) is a highly dynamic, complex and challenging clinical setting, and adding a SAR to this context requires a carefully designed process involving the healthcare providers (HCPs), patient, and caregivers throughout the development and deployment process.

The successful deployment of a SAR in a public space, particularly in the hospital setting, is a complex endeavour that demands long-term planning and meticulous execution. It must be designed with all involved participants, and their voices must also be heard on an ongoing and sustained basis; we must ask ourselves how to transfer the necessary knowledge in the right way according to each person’s skills and level of involvement, and even more importantly, how to ensure that their feedback is echoed at each stage. To achieve the above premise, the planning and overall project management must be agile and flexible; precise communication, validation, and evaluation mechanisms must be established, accompanied by specific technical tools, such as training, field notes and an efficient log reporting system. This is even more relevant in hospital settings and, especially, in an ED setting, where the site’s daily routines and the HCPs’ time commitments can change dramatically depending on acute external demands. This could put the successful execution of the project at risk, affecting not only the research team but also patients and caregivers.

We have developed an AI-enhanced SAR that is suitable for deployment in the EDs of two large tertiary care children’s hospitals in Canada. The robot has been designed to serve as a tool to distract children undergoing intravenous insertion (IVI), adapting to the child’s affective state and the course of the medical procedure. While the system is currently undergoing a clinical trial to evaluate its impact on patient outcomes, the present paper is not intended as a report of that trial design or its results. Rather, we focus on documenting the process of designing, developing, and deploying the robot system, which involved intensive, long-term collaboration among technical team members, clinical researchers, as well as front-line HCPs (including physicians, nurses, and child life specialists) and family members from the two hospitals. Detailed accounts of such processes are rarely published, yet they are essential for enabling replication, adaptation to other contexts, and informed methodological choices in future work with SARs. With that goal in mind, we describe the process in full detail, including technical specification of the robot system as well as detailed accounts of the procedures that were developed to allow it to be deployed. At the end, we discuss lessons learned at all levels from the deployment process, distilling those lessons into concrete guidelines for future work in this dynamic and challenging research context.

## 2 Related work

### 2.1 Socially assistive robots in healthcare

Socially Assistive Robots (Feil-Seifer and Mataric, 2005) provide a unique opportunity to use human-like social interaction to support embodied interaction to provide concrete assistance to the user. This type of Human Robot Interaction (HRI) is considered potentially helpful for creating a shared relationship by using human characteristics such as expressiveness, personality, dialogue, empathy, and adaptation skills for the robot design. Numerous SAR studies have reported benefits in various domains, such as social, behavioural, physical, and cognitive wellbeing in different populations (Amirova et al., 2021; Henschel et al., 2021), in applications such as robot-assisted education (Johal, 2020), autism diagnosis and therapy (Scassellati et al., 2018; Gomez Esteban et al., 2017; Pennisi et al., 2016), and Alzheimer and elder care (Tapus et al., 2009; Wada et al., 2004).

Paediatric healthcare is an increasingly active deployment context for SARs (Dawe et al., 2019). While some studies have investigated long-term robot deployments in contexts such as cancer wards (Ligthart et al., 2018; Moerman and Jansens, 2021; Logan et al., 2019), a particularly promising context is using the robot with the goal of reducing acute pain and distress in patients (Trost et al., 2019; Moerman et al., 2018). Several studies have compared robot-delivered distraction to standard care in the context of needle-based procedures, with generally positive effects. Ali et al. (2020) found that the addition of a robot reduced distress and pain compared to standard care in the context of needle-based procedures in an ED. In a similar study, Smakman et al. (2021) found that a robot reduced pre-procedure pain and stress for a subset of the children, while Rossi et al. (2020) also found a similar result for children undergoing vaccinations. In a study focusing specifically on using a robot to alleviate pain-related memory bias in children with chronic illness, Rheel et al. (2022) found positive clinical results as well.

Rather than directly comparing outcomes with and without the robot, other studies have examined the impact of different robot behaviours. For example, Jibb et al. (2018) compared user responses for a robot that used cognitive behavioural therapy to one that used just distraction (i.e., dancing): the children did not experience different levels of pain, but there was less stress in the distraction condition. In addition to measuring the impact of the robot, Rossi et al. (2020) also found that adapting the robot’s behaviour to the child’s perceived anxiety level had a positive effect on distraction. Trost et al. (2020) compared two versions of their robot, one that used scripted responses mimicking empathy and one that used pre-scripted dialogues aimed at distracting the patient: while they reported no significant difference on the mean scores of pain and distress, they did suggest that an empathic SAR could be clinically more effective since children were more willing to receive the medical procedure in this condition.

In a very recent systematic review, Pan et al. (2024) carried out a meta-analysis of randomised controlled trials of SAR interventions to reduce pain and distress in paediatric needle-based procedures. Across ten trials, involving a total of 815 children, they found that the SAR interventions significantly reduced anxiety and distressed avoidance behaviours, although the effects on pain and fear were not statistically significant. These findings highlight the promise of SARs to help in this context, while also indicating that further high-quality randomised controlled trials are needed to further validate the findings.

A significant limitation in most of the previous studies is that the robotic system autonomy has been extremely restricted: most have used fully scripted behaviour or a Wizard-of-Oz approach (Riek, 2012), where the robot behaviour is largely controlled by an experimenter. Indeed, restricted autonomy was identified as a key limiting factor in a recent survey of social robotics in the clinical context (Trost et al., 2019) and is one that we explicitly address by deploying a robot that is able to interact autonomously with the patients in a real-world setting.

### 2.2 Consultation of interested parties for public-space robotics

Although much of the initial work on social robots was lab-based, an increasing number of social robots are now employed in public spaces (Mubin et al., 2018; Schneider et al., 2022). For example, Pepper robots have been deployed in a wide range of contexts including shopping malls (Foster et al., 2016), museums (Daczo et al., 2021), restaurants (Stock and Merkle, 2018), libraries (Mubin et al., 2020), and train stations (Thunberg and Ziemke, 2020); recent deployment roles for Furhat robots have included a barista (Lim et al., 2022), a receptionist (Moujahid et al., 2022), and a worker in an university student centre (Ashkenazi et al., 2024); while in Japan, Robovie robots have been successfully used for a series of shopping mall deployments (Kanda et al., 2010; Shiomi et al., 2013; Glas et al., 2016; Brscic et al., 2017; Shi et al., 2017).

A common factor in all these deployment contexts is that they are existing workplaces, with interested parties including management, visitors (patients, customers, tourists), and front-line workers who should all be consulted to develop the details of the system to be deployed (Niemelä et al., 2019). The managers are clearly influential: no deployment can be effectively carried out in such a space without the permission of the management, who also exercise significant control over the time and place of the deployment, as well as the topics that the robot might discuss and how it engages with visitors. When it comes to the building visitors, who are usually the target end-users of any such deployed robot, user-centred design processes are common in the HRI community (Charisi et al., 2016; Thunberg and Ziemke, 2021), and the success of most interactive robot systems is also usually assessed through studying user behaviour and subjective responses (Apraiz et al., 2023).

Front-line workers—in our scenario, HCPs in EDs—need to continue to do their jobs alongside the robot during the deployment and may even be called on to provide hands-on technical support for the robot, especially if the deployment site is remote from the developers (e.g., Foster et al., 2019). Historically, it has been rare for these key invested personnel to be considered either in the design or evaluation processes, despite their needs and opinions being paramount to the overall success of deploying social robots in their work environments. For example, when describing a study conducted in a German elderly-care facility, the authors note: *“In spite of quite extensive discourse with the facility management prior to the on-site experiments, not all staff and residents were involved in the preparation, which may have led to some issues of the staff not giving full consent”* (Langedijk et al., 2020, p. 790), while in a recent meta-analysis, nurses expressed similar concerns about care-home robots (Trainum et al., 2024). During a previous robot trial in the emergency department, approximately one in three robot deployment events had a technical issue (Ali et al., 2020), while the nurses involved in a recent clinical trial of a robot also identified multiple challenges and disadvantages of the system (Rheel et al., 2022). In our work, we have explicitly included all of these personnel throughout the design and deployment process to ensure the success of the ultimate robot deployment.

## 3 Materials and methods: the robot system

We have developed a fully functioning companion robot^1^ for operating in the IVI scenario, which was built using both a co-design phase (involving several iterative cycles with children, parents, and HCPs) and targeted meetings between the technical team and the HCPs. The robot is designed to work alongside an HCP throughout the key stages of the IVI procedure: introduction, pre-procedure, optional site-check, procedure, debrief, and conclusion. A key aspect of the system is that, unlike previous SARs used in similar deployments (e.g., Trost et al., 2019; Ali et al., 2020), the interactions are customised to the patients and their specific pathways through the procedure, using AI planning (Ghallab et al., 2004) to choose autonomously among the different robot behaviours based on the system’s estimate of the situation.

**FIGURE 1:**
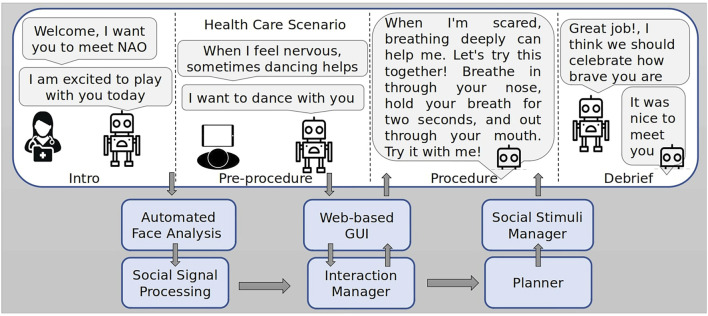
System architecture including social signal processing, interaction management, and AI planning system (Lindsay et al., 2024b). Robot utterances are based on Ali et al. (2020).

The full system (Figure 1) is composed of several components, including social signal processing, an interaction manager, and an automated planning system. It is implemented on the NAO robot platform, supported by embedded hardware (a Raspberry Pi 4 and an nVidia Jetson GPU), a Sterolabs ZED 2 camera, and a tablet running a web application for operator control, using the Robot Operating System (Quigley et al., 2009) for integration. The system makes full use of NAO’s 25 degrees of freedom, combining body language, gestures, and speech. During the interaction, the robot performs behaviours such as dancing to music, suggesting strategies for the child to use during the procedure, and asking questions directly to the patient. In this section, we present the co-design process that we followed to design and implement the robot system and give details of the technical components of the system. This system was the starting point for the process of consultation and deployment that we report in detail in the following sections of the paper.

### 3.1 Co-design steps

**FIGURE 2:**
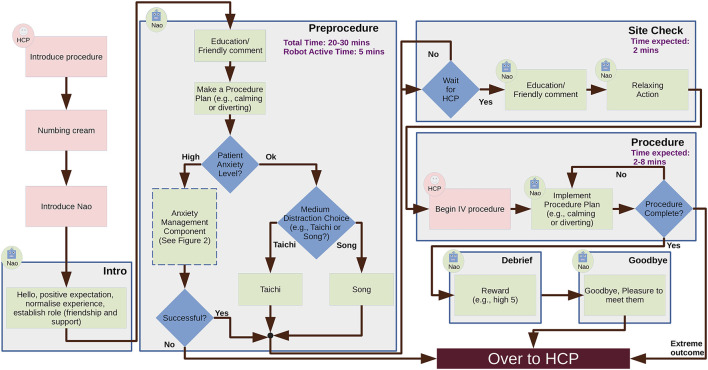
Flowchart illustrating a possible IVI scenario incorporating HCPs and a SAR.

**FIGURE 3:**
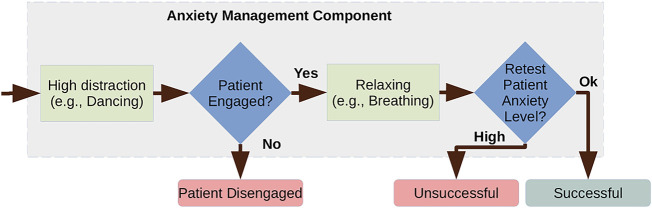
Anxiety management component.

To model the IVI domain, a two-cycle co-design study was conducted (Nishat et al., 2023; Hudson et al., 2023). The study consisted of a qualitative assessment of the needs of HCPs in two paediatric EDs. Semi-structured virtual individual and focus group interviews were conducted with 11 health professionals (5 nurses, 4 physicians, 2 child life specialists), who had varied years of experience in paediatric emergency care (1–21 years). In the second cycle, multiple workshops were held between the technical team and clinical staff to analyse and interpret the findings of the focus groups and to turn them into technical requirements for the HRI system. As a result of the participatory process, a series of guidelines for the design of the intervention emerged. In addition, a flow chart was also generated (Figures 2, 3), gathering the indications generated and showing the interpretation from a technical perspective, highlighting the most relevant features for the entire team.

In the first co-design cycle, predicted benefits of the robot-based intervention were specifically classified based on the main phases of the IVI procedure (before, during, and after), as follows. Before a pain-related procedure, HCPs stated that the ideal aims of the robot intervention are to provide emotional support and education, and to promote coping strategies. During a procedure, participants identified a change in the primary aim, i.e., the robot could behave as a distraction based on the child’s anxiety and engagement level and adapt to the child’s age-related preferences. Following the procedure, HCPs emphasised the importance of framing the medical procedure experience as positive for the child through positive reinforcement, rewards, and debriefing conversation by having the robot remain to support the child.

With regards to robot behaviours, HCPs felt that the proposed SAR should be equipped with a diverse range of actions to suit children’s needs, including encouraging dialogue, positive reinforcement phrases, humour, and cognitive behavioural strategies (e.g., breathing techniques, guided imagery, meditation). HCPs also felt that the proposed SAR should allow the user (i.e., child) to choose from a selection of options for distraction. In addition, the proposed SAR should also have the ability to estimate social signals and generate responsive social stimuli accordingly as children exhibit affective states (verbally, physically, and emotionally).

At the end of the first cycle, HCPs highlighted two main constraints on system reliability. First, it must never speak over an HCP while information is being delivered; in other words, the system must maintain proper turn-taking between the HCP and the robot. Second, the proposed SAR must not act inappropriately. This includes both emotional insensitivity (e.g., telling a joke when a child is crying), as well as situations where there is a lack of awareness of clinical deterioration (e.g., seizures, loss of consciousness): in these scenarios, the HCPs emphasized the importance of being able to immediately pause the SAR or turn it off entirely.

Following the first cycle, two workshops were held, during which the team’s clinical and technical specialists, together with four additional HCPs (3 nurses and 1 child life specialist), discussed the previous focus groups’ findings from a technical perspective. As a first step, most of the HCPs suggested that the entire IVI procedure be divided into six steps. Subsequently, an objective related to psychological exercises to reduce distress was assigned in each stage. This is a distinctive aspect of the current scenario compared to the previous robots discussed in Section 2.1, as it explicitly changes the role of the robot as the procedure progresses. Then the whole group defined the role of the robot at each stage, the plausible activities for each role, and their relationship with the child’s emotional state. Likewise, the total time of the stage and its distribution between the HCP and the SAR were established. The result of the discussion is summarised in the technical requirements listed in Table 1.

**TABLE 1:** Findings translated from HCP view to technical HRI requirements.

Stage		Intro	Preprocedure	Site check	Procedure	Debrief	End
HCP Requirements	Psychological exercises	Positive expectation	Coping exercises	Normalize experience	Distract	Positive reinforcement	Form positive long-term memories
	Stage Goal	Engage the Child	Manage anxiety keeping engagement	Keep Engagement	Divert	Re-engaged the Child	Inform/Educate
	Robot Role	Robot Peer	Robot Tutor	Robot mediator	Robot Assistant	Robot Peer	Robot Interviewer
	Timing	HCP: 2–3 min R:2–3 min	HCP: 10–15min R:5–10min	R:2–3min	R:2–8min	HCP: 2–3 min R:2–3 min	HCP: 2–3 min R:2–3 min
Technical Requirements	Planning Goal	Establish role Set up positive expectation	Manage anxiety Divert Make plan for procedure	Re-enforce strategy Calm Keep patient still	Implement diversion plan Respond to delays	Congratulate Reward	Recount key points Finish interaction
	Metrics	Attention Level	Attention and Anxiety Level	Attention and Anxiety Level	Anxiety Level	Attention Level	Attention Level
	Data Sources	Gaze Direction	Gaze Direction Head/Facial features User Input/queries Child choice	Gaze Direction Head/Facial features User Input/queries Child choice	Head/Facial features User Input/Queries	Gaze Direction	Gaze Direction

### 3.2 World state estimation

**FIGURE 4:**
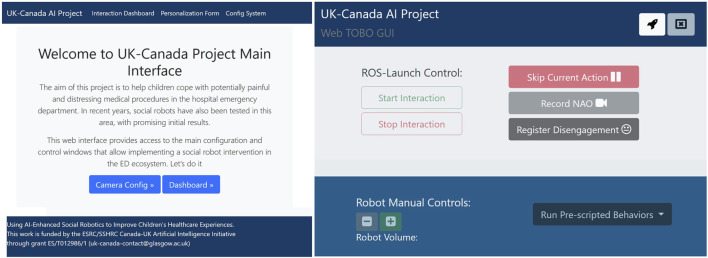
Web-based Interface used to control the system and to confirm social signal processing hypotheses. On the Right, the welcome page, on the left, the main control dashboard.

In the early stages of the co-design process, the participants identified the need to integrate a social signal prediction module, as the most important form of behaviour adaptation in this context involves monitoring and responding to the state of the patient. However, due to the inherent complexity of the IVI procedure, the uncertainty of the social predictions, as well as the identified need for reliable and robust behaviour, it requires special care to ensure the correct interpretation of the scenario. Therefore, we designed a world state estimation module that combines predicted social signals based on sensor data together with explicit feedback given by a research assistant about the patient’s emotional state, engagement, and willingness to participate in the procedure; the tool also allows the researcher to control the steps of the procedure and to stop the interaction entirely, if necessary. The interface for this tool is shown in Figure 4. The interface initially displays a welcome message that briefly introduces the project. From the welcome page, users can navigate to any of the developed subpages using the top menu bar. On the left of Figure 4, the main control page is shown, where users can perform several functions in addition to those mentioned earlier. These functions include adjusting the volume, monitoring the battery level, recording moments of interaction that they find significant, and noting instances of the patient’s disengagement. Additionally, this main page generates pop-up messages to confirm the child’s emotional status, as well as their preferences and personalisation choices.

#### 3.2.1 Face analysis pipeline

The core component of the world state estimation process is predicting the patient’s affective state. Due to the physical constraints of the robot deployment, the main automated mechanism that is available for this setting is facial analysis based on the camera data. Automated prediction of the patient state in this setting is challenging mainly due to occlusion: the patient will likely be wearing a surgical mask, and there may be a constant flow of staff in the room. Additionally, the use of the space close to the patient is limited, which means that the equipment (e.g., the camera) must be located at some distance. Internet use is also limited by interference generated by the high flow of devices using the wireless network in the room.

With these limitations in mind, a pipeline has been developed to automatically analyse the patient’s face, providing hypotheses which can be confirmed by a researcher through the web interface. The automatic analysis pipeline is based on Nvidia DeepStream SDK (Nvidia, 2022) and was deployed using a Jetson Nano board. For the low-level face analysis behaviour pipeline, we used the FaceX-Zoo framework in the face and landmarks detection stage (Wang et al., 2021). We selected two models, a PyTorch implementation of the RetinaFace model (Deng et al., 2019) and the Practical Facial Landmark Detector (PFLD) (Guo et al., 2019). These models were retrained using the MegaFace-Mask database, improving the detection of subjects wearing a mask.

**FIGURE 5:**
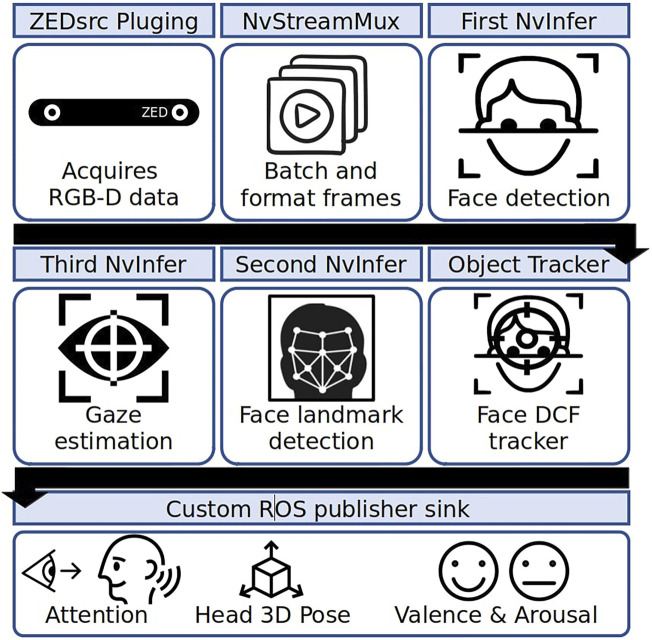
Low-level face analysis pipeline.

Taking advantage of the ready-to-use hardware-accelerated plugins, we used TensorRT (Nvidia, 2025b), Nvidia’s inference accelerator runtime, for model inference. In addition, we used built-in plugins such as the Nvidia-adapted Discriminative Correlation Filter (DCF) tracker (Lukezic et al., 2017) and the EmotionNet and GazeNet inference. EmotionNet (Nvidia, 2024) is a classification network with five fully connected layers and was trained on MultiPie dataset to classify six emotions. GazeNet (Nvidia, 2025a) detects the patient’s gaze vector and point of attention and was trained on an Nvidia proprietary dataset. As a final element of the pipeline, our system uses the Point Distribution Model (PDM) from the OpenFace toolkit (Baltrusaitis et al., 2018) to calculate the 3D head pose, and a ROS-based plugin to publish the features estimated along the pipeline, including the patient’s visual focus of attention. The plugins implemented in the pipeline are shown in Figure 5; each individual block represents a specific plugin, which are together optimised through memory management with zero-memory copying between plugins, ensuring efficient performance.

The low-level face analysis behaviour pipeline is responsible for detecting the patient’s face with a mask, identifying facial landmarks, head pose, gaze direction, and facial expression. Based on the above facial features, the social signal processing module estimates the current focus of attention and the head movement speed. This information is used to estimate the patient’s focus of attention and emotional state, providing an indirect measure of affective states such as anxiety, valence, arousal, and engagement, which are needed to control system behaviour. The predictions of this model are constantly published to the interface, where the user can either confirm or override the estimated world state as necessary.

Although the affective state pipeline, as described, performs robustly in lab conditions, we have adopted a conservative approach to using our pipeline in the deployed setting. Along with its predictions, the system predicts a confidence value (0–1), which is used with a threshold to determine whether the predictions have sufficient confidence. These predictions are used to provide recommendations for the patient’s affective state. However, the operator has final control over the predictions used by the system. This conservative approach was adopted because our aim in this work is to investigate the use of the patient’s affective state in generating appropriate behaviours during the procedure. Our approach allows us to build up data, ultimately leading to an improved model, while also being more confident that the robot’s behaviours are being generated based on robust predictions of the patient’s affective state.

### 3.3 Plan-based approach to managing affective state

**FIGURE 6:**
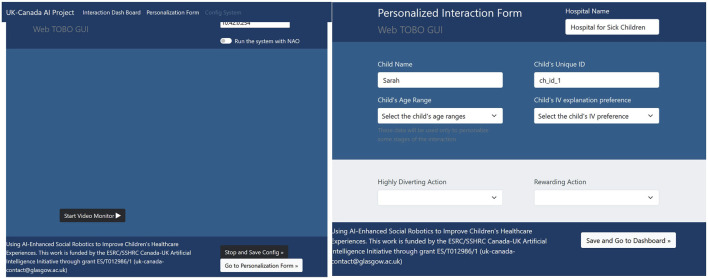
Web-based interface used to personalise the multimedia content and configure the video recording pipeline.

In our approach, the robot’s behaviours are underpinned by an AI planning model, which allows us to capture the rules of the interaction. To specify the model, we began with the interaction sequences presented in Figures 1, 2, and then generalised the interaction for different patient pathways and medical outcomes. Taking as input the world state estimation (see above), the planning system generates the most appropriate sequence of robot behaviours, customising the interaction for the specific patient and scenario using the user’s own preferences collected using the web interface shown in Figure 6. For example, Figure 8 presents different pathways for a patient with high anxiety, which is subsequently managed by the system (left), and a patient who is anxious and disengaged (right).

**FIGURE 7:**
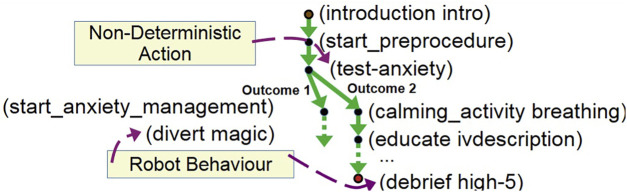
A partial plan showing actions (e.g., breathing exercises and high five), sensing actions (e.g., testing patient anxiety) and procedure actions (e.g., the preprocedure start).

The planning model uses a declarative representation to encode the domain knowledge and possible interactions concisely. We use a fully observable non-deterministic (FOND) planning model based on the work of Muise et al. (2012), which can be defined as a tuple ⟨F,I,G,A⟩ , with fluents F , initial state I (a full assignment to F ), a partial goal state G , and a set of actions A . Each action a∈A is a pair $\left\langle {pre}_{a},{e\mathrm{ff}}_{a}\right\rangle$ , with a precondition ${pre}_{a}$ (a subset of F that must hold) and an effect ${e\mathrm{ff}}_{a}$ (a set of possible outcomes—fluents that are made true or false). If an action defines one outcome, it is a deterministic action; otherwise, it is a non-deterministic action. Each action application results in an outcome, but the outcome cannot be chosen by the planner. A solution to the problem is a branched plan π (see Figure 7), which includes alternative action outcomes and describes the sequence of actions that will achieve the goal, given any outcome.

#### 3.3.1 Incorporating sensing of affective state into the planning model

In our medical scenario, a key concern for the HCPs during the procedure is that patients typically harbour anxiety inducing negative anticipation and uncertainty about the procedure. The robot therefore makes a plan of how the robot will support the child during the procedure and provides the child with certainty. The aim is to reduce negative anticipation and uncertainty by replacing it with positive anticipation. We could attempt to try to capture the impact of anxiety on the user provided by this support. However, it is likely that the impact of this strategy will be fairly subtle across the interaction, making it challenging to model as an effect and impractical without substantial data.

As an alternative, we can consider incorporating sensing into the model, which allows the uncertainty of particular outcomes to be explicitly represented (such as the outcome of the test-anxiety action). For each aspect of affective state, we define a variable and an appropriate set of values. For example, we define a variable $v_{\mathrm{anxiety}}$ , with normal and high values. The test-anxiety action defines an outcome for each of the possible values, and sets the appropriate value of $v_{\mathrm{anxiety}}$ in the state. The value of the affective state variables can be used in the action preconditions, and consequently the patient’s affective state helps determine the subsequent robot behaviours.

Figure 7 presents part of a branched plan, which includes the sensing action test-anxiety. This allows the plan to capture the strategy in the case of either high (e.g., selecting an appropriate intervention) or normal anxiety (e.g., continuing the interaction by practising breathing exercises).

**FIGURE 8:**
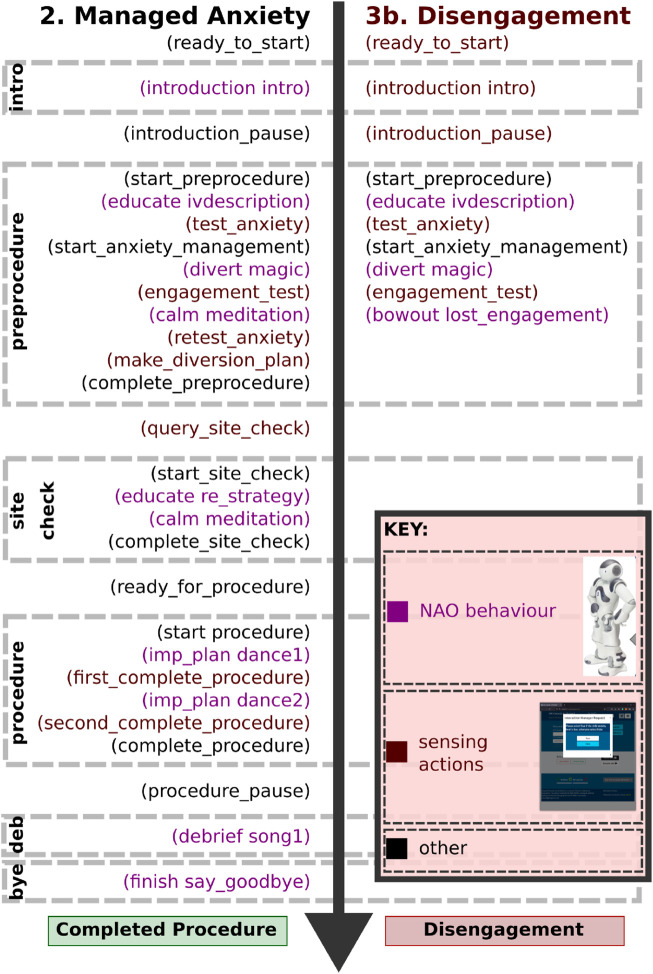
Actions from partial plans showing robot behaviours (purple) and sensing actions (red).

The planning model allows new state information to be interpreted within an appropriate context. In our medical scenario, we can monitor engagement during the interaction, and cases of disengagement can be indicated by changing the robot’s state representation. We defined a variable for engagement, $v_{\mathrm{engaged}}$ , with values of OK or low-engagement. The robot’s response to disengagement is determined based on the context. For example, if the patient becomes disengaged while also highly anxious, it is important that the robot stops its interaction, to prevent worsening the situation with additional noise and an unwanted voice (e.g., see Figure 8, right). However, in more typical scenarios, the robot can choose an appropriate action in order to attempt to re-engage the patient, such as encouraging their interaction and giving them options between different songs, or other robot behaviours.

#### 3.3.2 Anxiety management component

It is already clear that there is a potential for social robots to have a positive impact on human affective state (e.g., Jibb et al., 2018), however, it is also apparent that we do not fully understand some of the interactions that occur during human-robot interaction. As a result, it is important that the robot’s actions during the interaction are carefully monitored to ensure that its behaviour is making the intended impact. This is particularly important in situations with vulnerable users, such as our context, where inappropriate action may lead to harm or distress.

In such cases, we adopt a strategy that consists of: testing, manipulating and retesting. The aim is that a first test is used to identify any issues (e.g., an aspect of the user’s affective state), and determine the appropriate course of action. As part of our design process, we have identified specific points during the interaction (e.g., after the HCP has explained the procedure) where the patient might become anxious. The anxiety test action (a sensing action that determines whether the patient’s anxiety level is OK) is used to determine the patient’s level of anxiety, and in cases where the patient has high anxiety, we adopt an anxiety management procedure.

The anxiety management procedure combines certain specific interventions with monitoring of social signals, with the aim of managing the patient’s anxiety. An example intervention adopts a strategy of first diverting with a high-distraction activity, such as dancing, and then attempting to calm the patient, using a relaxing activity, such as breathing exercises (see Figure 8, left). The system predicts that the patient’s anxiety will have reduced by this procedure, and this prediction is confirmed by retesting the patient’s anxiety level at the end of the procedure.

## 4 Consultation and real-world usability testing

The ultimate goal of the project was to deploy this robot system in two large children’s hospitals and carry out a formal, two-site, randomised controlled trial of the robot in the real world, comparing perceived and reported pain and distress between IVI patients who interact with the robot with those of patients who received standard care. To prepare for that trial, extensive technical validation, training, and usability testing were conducted at both clinical sites to ensure that the robot and associated processes were ready for trial use.

**FIGURE 9:**
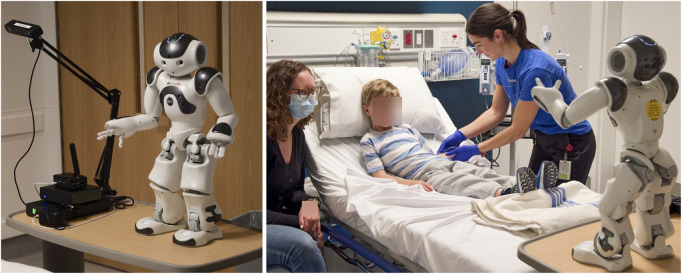
Real-world robot hardware and deployed scenario (images from SickKids Simulation Lab).

While consultation was a key aspect in developing the original version of the robot, as described in Section 3.1, additional consultation was also a crucial aspect of translating the original version of the robot system into a version which could operate in the two deployment locations, and—critically—that would integrate into the clinical processes in a way that would allow the formal clinical trial to be carried out. Figure 9 shows the final robot hardware and an illustration of the deployment context.

**FIGURE 10:**
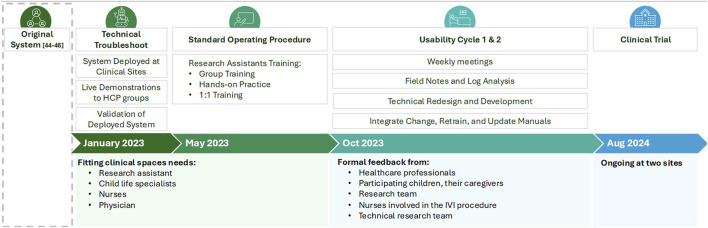
Long-term iterative process of the design and deployment of the SAR in the ED.

We built on the initial co-design sessions as part of the deployment process, which was structured as a long-term iterative process with multiple stages of feedback, redesign, and testing (see Figure 10). The process began when the original version of the robot system was made available for deployment. The following steps included technical validation at both clinical sites, followed by the development of a Standard Operating Procedure (SOP) for personnel at both sites to operate the robot. Following that, a two-cycle usability trial was carried out to ensure that the robot was ready for its final deployment, and that personnel at both sites were ready to support the deployment. The process ended when the system was deemed ready to undergo the formal clinical trial. This sequence of steps was of utmost importance in the current healthcare setting, as it ensured that the technology was correctly integrated into the clinical workflow and that knowledge was effectively transferred between the participants involved, considering their expertise and level of involvement.

### 4.1 Technical testing and standard operating procedures

When the clinical research teams first received the robot system in January 2023, the initial step was to test it in an ED clinical room in the target locations. The teams first verified that the robot and necessary equipment fit into the available clinical space. Once confirmed, the teams carried out a range of technical tests to ensure that the robot was ready to be deployed with patients. As part of this verification process, we utilized several methods to gather clinical input, including consulting with child life specialists and nursing staff to ensure the robot’s dialogue scripts contained appropriate and accurate language. We also provided live demonstrations of the robot at ED nursing stations and physician staff meetings to gather meaningful input from clinical staff.

The robot system was also tested prior to beginning usability testing to ensure actions and movements were smooth and easy to understand. Several identified areas of improvement (i.e., adjusting the volume for songs, cutting the length of the audio files, and removing commercial behaviour) were documented based on assigned priority levels and communicated to the technical team to make revisions. The technical team addressed all critical and high-priority items, followed by retesting by the clinical team.

Once the robot system was considered sufficiently reliable for usability testing with patients, each site developed SOPs with photos and step-by-step instructions to follow. The training was completed with the ED research assistants and coordinators, who were responsible for recruiting patients and operating the robot system with patients. In-person training was conducted in the form of a single 1.5- to 2-hour session, offering hands-on practice and one-to-one refresher training upon request to ensure research assistants could all operate the robot system in the ED and troubleshoot any minor technical issues independently. The full technical testing process ran from May through October 2023.

### 4.2 Usability cycle feedback

After the system was validated and SOPs agreed upon, we began real-world usability testing. Ethical approval was obtained from the ethics committees of both clinical sites as well as the technical partners’ ethics committees.

Feedback from all participants was crucial during the ongoing development and knowledge transfer phase. We gathered input from HCPs, participating children, their caregivers, and the clinical team. Research assistants and ED nurses involved in the IVI procedure also provided detailed feedback to ensure that all perspectives were considered.

#### 4.2.1 Usability testing

The usability testing stage involved weekly meetings between the clinical research coordinators and the research team responsible for technological development. Additionally, we undertook retraining and validation sessions to ensure that the changes made were effectively integrated. These sessions were conducted closely with the HCPs and clinical team to ensure their interactive participation. This stage spanned approximately 10 months, commencing in October 2023 and concluding in July 2024 after the release of the final stable version of the system.

Usability testing was carried out in two iterative cycles with patients and their caregivers at both clinical sites. For each cycle, the robot system was deployed during an IVI procedure, and the interaction was video-recorded and observed by the clinical research team, followed by a brief satisfaction survey and a semi-structured interview with the child and caregiver. Both cycles included video recordings of the intervention, field notes, and interviews with all children and their caregivers to identify primary and minor issues and potential enhancements. Feedback from the HCPs, research assistants, patients, and caregivers was captured via field notes and observation. Based on feedback, adjustments were continuously made to improve the robot system; small adjustments were made during each cycle, while larger technical changes were made between the two cycles. The details of the technical update process are given in the following section. The second cycle continued until the point where no new information was gleaned from new sessions.

Across the two cycles, a total of 25 children and their caregivers were included in usability testing. Table 2 shows the demographic information of the included children.

**TABLE 2:** Demographic information from studies.

	Cycle 1	Cycle 2	Total
Site			
Site 1	11	8	19
Site 2	2	4	6
Sex			
Female	10	5	15
Male	2	7	9
Missing Sex Information	1	0	1
Age (years)			
5	2	3	5
6	3	2	5
7	1	1	2
8	2	2	4
9	1	1	2
10	0	2	2
11	2	1	3
Missing Age Information	2	0	2
Total	**13**	**12**	**25**

#### 4.2.2 Results of first cycle

Feedback from the first usability test cycle consisted of four aspects: technical issues, usability issues, positive feedback, and feature requests. Table 3 summarizes the items mentioned in the context of each aspect; here, we discuss each aspect further.

**TABLE 3:** Common issues detected after the first usability cycle.

	Common issues detected
Technical Issues	- RGB-D Camera didn’t work reliably - On occasion, NAO robot didn’t stop when asked - The robot repeated a song - The robot continued the dance without playing the song - The robot played the song without moving - The personalised content was not played
Usability Issues	- Long pauses disrupted the flow of the intervention - The robot intervention was faster than the IV procedure - The robot intervention was slower than the IV procedure - Some patients preferred not to hear any language about a needle or poke - The volume level of the songs was not appropriate - Patients seemed confused by some of the dialogue
Positive Feedback	- Patients liked the robot’s voice and dance moves - Nurses were excited and receptive to the robot intervention - Patients and caregivers liked the personalized content - Caregivers described how it took the pressure off them to distract the child
Wish List	- More popular songs by age - More personalized content - More friendly user interface elements

##### 4.2.2.1 Technical issues

Research assistants felt that the dashboard interface was initially not intuitive, but the training and practice helped them gain confidence in running the robot system; their confidence was also increased by interface enhancements driven by feedback from the first usability cycle. Among the main technical issues, they identified problems with connecting to and streaming the input from the RGB-D camera. Some minor issues included the robot not stopping the interaction when indicated, showing repetitive behaviours using the same multimedia content, and glitching without sound or movement. The families also noted that the music they had chosen did not play during the IVI procedure.

##### 4.2.2.2 Usability issues

Among the usability issues that were noted, excessive pauses were identified in the transition between IVI stages and poor synchronization between the IVI procedure and the intervention with the robot: sometimes, the IVI procedure was slower than the robot, and other times, the opposite occurred. Additionally, there was not enough personalization of the content, especially regarding the language related to the IVI (“needle”, “poke”) or whether children desired to hear an explanation. Some phrases from the robot were deemed ambiguous or confusing.

##### 4.2.2.3 Positive comments

Among the positive comments, it is notable that the robot system was described as being fun to interact with, and a few caregivers described how it took the pressure off them to distract the child during the IVI. Despite some technical difficulties, families and HCPs expressed positive feedback regarding the robot’s ability to distract the child during the IVI procedure.

##### 4.2.2.4 Feature requests

Finally, HCPs and patient partners suggested more personalization based on the child’s age and their preference for hearing an explanation about the IVI procedure. Following this, a wish list was added that included popular songs and movements according to the age of the patients, as well as rewording some parts of the content and options to skip the explanation of the IVI procedure.

#### 4.2.3 Results of second cycle

For the second usability cycle, research assistants felt that the operation of the robot system was perceived as more intuitive, and they had more confidence in the function of the robot as intended (noted fewer glitches and unexpected pauses). Research assistants were able to use the added features to allow more personalization for each child while following a simplified interface with less room for error. The patients and families continued to note positive experiences with the robot. HCPs continued to be supportive and noted the importance of close communication between the clinical and technical research teams to ensure adequate time for setting up the robot system.

### 4.3 Ethical considerations

The consultation phase also included questions regarding ethical aspects of the robot design as well as the interaction design (Zeller et al., 2021). From the ethics-specific questions during that process, which related to privacy needs and trust, it is clear that the relationship of the caregivers with the hospital and HCPs formed an important factor in the overall trust of the robot. In fact, some feedback showed that caregivers’ trust in HCPs extends almost automatically to the robot. Other caregivers connected trust in the robot system to the actual goal of the robot. For example, it was stated that AI in the form of a robot learning about the individual child was fine as long as it was for an improved interaction with the child. In the case of the robot system collecting data merely for learning in general, then less support was expressed. Relevant outcomes on the side of HCPs were primarily addressing the procedure impact, that is, worries about whether using a robot would extend the overall time for the actual procedure, which is a significant factor in an ED environment. On the other hand, Zeller et al. (2021) also found indications towards overall approval of a robot to help with distraction and pain management. Overall, these ethics-related outcomes emphasised the need to actively include all relevant participants in the design and use of a SAR. Furthermore, they underscored the importance of close collaboration between the HCPs and the technical research team.

## 5 Feedback from the research teams

Once the consultation process had finished, the technical and clinical teams were able to reflect on their experiences during the process.

### 5.1 Technical research team feedback

Due to the nature of the deployment, it was not appropriate for the technical team to be on the ground during deployment. As a consequence, there was a separation between the observation of the robot system in deployment and the location of the research technical team, which could diagnose issues and update the system. However, we implemented a direct communication process that made the technical team always accessible. This approach allowed us to minimize the time from issue identification to resolution. The process for recording an issue involved the operator (research assistant) gathering a video of the interaction, providing a detailed error description, and recording their observations and those of the HCPs in field notes. Once the issue was documented, the clinical research team broadened the context through direct conversations and additional observations and transmitted the collected information to the technical research team via email.

We used the information gathered, such as system logs, field note comments, and the details of the context, to pinpoint and diagnose the potential source of the problem. Weekly meetings were held between the technical team and clinical research coordinators to review the issues detected during the week and make necessary decisions for resolution. Depending on the problem type, the solution could range from updating the operation guide and adjusting the training of the operators to prevent mishandling, to fixing code bugs or re-implementing software modules using a remote access mechanism. We established secure remote access and control mechanisms to address issues using traditional tools, efficiently track software changes, facilitate collaboration, and seamlessly update the software.

#### 5.1.1 Directly actionable changes

The most straightforward issues involved comments or requests that corresponded directly to actionable changes in the system. For example, it was noted that there was insufficient personalised content and that it was not appropriately organised for age groups. From a technical point of view, we made a new system to categorise the robot’s content (songs, etc.) in terms of its appropriate age range and the degree to which it was calming or diverting. We then categorised the existing content and added new content based on recommendations made during the usability testing. These categories allowed us to offer appropriate content in a more effective manner while also making better choices for the content during the interaction.

Another example of directly requested changes involved the incorporation of indicators and controls to directly oversee intrinsic components, such as the sound’s volume and battery levels. Although these components are essential to any robotic system, they are not always readily accessible to end users through a simple interface. Given the complexity of the scenario, operators requested direct access to these components through intuitive buttons on the user interface.

#### 5.1.2 Identifying behavioural patterns

Several of the issues required further examination of the observations, and for behavioural patterns to be identified. For example, in the case of both the personalised content not being properly used and camera initialization issues, it was discovered that during preparations for the intervention, the operators were using the interface in an unintended way. Initially, the interface provided operators with the flexibility to navigate between various pages for tasks such as configuring the camera, customizing content, and accessing the main dashboard to initiate the intervention. However, after studying the research team’s usage patterns, it was determined that restricting access to certain buttons and navigation menus or removing them entirely was necessary to create a streamlined and more intuitive operational flow.

Another event during deployment made clear the importance of careful monitoring: in this case, the monitoring was necessary to ensure that the system’s operators fully understood the system and its use. The deployment of the system relies on the careful training of several clinical research assistants who operate the system on the ground in the emergency departments. It is common that users may develop strategies to meet their needs that do not match the intended use of the system, and we have observed this in this deployment.

During the procedure, the operator provides information to the system (e.g., the child’s anxiety level), which is used by the AI planning system to guide its behaviour selection. In certain situations, the system was designed to stop the interaction: in particular, situations where continuation would be inappropriate, such as during medical complications. However, due to a misunderstanding or miscommunication of the system’s intent, the operators had encountered situations where they were surprised that the system stopped the interaction. Consequently, the operators avoided certain pathways in some sessions, as a strategy to prevent these situations. By examining the system logs of the early tests, the technical team identified that the interactions never followed specific (expected) paths. We were then able to discuss this during our meetings, and through several sessions, were able to understand that the operators were uncertain of the intention of some of the system’s questions and the meaning behind some of the robot’s behaviours. As a result, we were able to clarify the wording of the operator queries on the tablet interface and provide the operators with clearer instructions regarding aspects of the robot interaction. We were then able to confirm from future logs that operator behaviour had changed.

#### 5.1.3 Looking beyond the reported issues

In the original system, the patient’s engagement was rarely considered, and only in situations where its use was essential to determine whether it was appropriate to continue the interaction. Unsurprisingly, due to the nature of the deployment within busy EDs, there can be frequent distractions, which can lead to the children becoming disengaged with the robot. Although it was not explicitly reported, we became aware that the operators were using the pause button on the interface more often than was expected. After the clinical research team investigated, it was discovered that in some cases the button would be used while some distraction was happening, and they had discovered that the button provided an effective workaround.

However, it was clear that there was an opportunity to provide a more tailored solution for managing patient disengagement, which could both take into consideration the context, and also adopt additional strategies for re-engagement. As a consequence, we designed and implemented an extension to the system so that either the social signal module or the operator can report disengagement at any time during the interaction. As a further advantage, the additional engagement information provides an additional layer of feedback for the interactions.

### 5.2 Clinical research team and HCP experience and feedback

From the perspective of the clinical research team, some important strategies were noted that helped with engaging HCPs and smoothly conducting the real-world usability test.

First, adequate training sessions and practice opportunities improved the confidence of research assistants who were directly communicating with HCPs and flagging eligible participants. We witnessed an improvement in their confidence between the two usability test cycles, from slowly walking through the steps to smoothly running the session and independently solving some problems. After, we noticed that a few notable research assistants were extremely helpful in conducting the usability testing cycles.

Second, the engagement of HCPs through multiple demonstrations of the robot was effective in the deployment process. The robot was brought around the nursing stations in the ED for demonstrations during clinical shifts. The robot was introduced to the physicians during a brief presentation at a staff meeting. The chance to see and ask any questions about the study allowed smooth integration of the robot system in the ED. Especially as one of the sites had over 200 HCPs working in the ED, the active engagement process helped with familiarizing them with the study. We were also able to identify HCPs who were champions of the study to seek support with recruitment.

The clinical research team also found that the weekly meetings with the technical team were helpful in discussing the process and working through suggestions from HCPs and families. The research coordinator at each site was able to act as a point of contact for research assistants to address all technical questions during the usability test.

Lastly, the feedback from HCPs, patients, and parents highlighted that personalization in the robot system is important as perceived experience on each session varied by child. The patient’s perception of the look of the robot and its activities especially varied in their comments. For example, some children thought it “looked good” and “liked how it looked” while some reported that they found the robot “creepy”. Some liked the song choices, while some wanted other songs. Overall, based on children’s past experiences and preferences, if given more time and resources, a future step would be to explore more personalization options to enhance the robot’s effectiveness.

## 6 Lessons learned

We can identify a number of key lessons learned throughout this process, including technical design considerations, effective communication strategies with all parties, and practical challenges encountered during the deployment phase. In this section, we outline some of these lessons learned during the project, which are also summarised in Table 4. At the end of the section, we use these lessons to derive a set of guidelines for future similar robot deployments.

**TABLE 4:** Summary of lessons learned during the consultation and usability testing phases.

Category	Item	Lesson	+/−
Team Participation	RA operators/HCPs involvement	Insightful feedback in terms of interaction, appearance, user interface (RA operators); Essential for getting patients/parents to trust robot	+
	Input on Robot Behaviours	(Put the effort in to) Enable full team participation where appropriate. For example, the full team participated in editing the individual robot utterances. This process led to excellent discussion, and huge improvements in the robot’s words	+
	(Re)Training RA Operators	Retraining allows flexibility in the interface and system during usability testing; Can help in uncovering misunderstandings	+
Communication	Weekly Meetings	Important for building common language between teams, to allow reactive system updates, and to ensure even small issues are discussed	+
	Identify Data Coordinators	Limit full team admin; Effective at organising and prioritising issues	+
Environment	Lack of Internet	Challenging for developing an interactive and social robot; Makes mundane tasks like data transfer challenging	−
	Sensitivity of environment	Lack of access for technical team to deployment location makes effective communication vital, e.g., small details regarding social interaction; This is particularly challenging in environments where video data is not available or very limited	−
System	Logging	Simplify and structure analysis of logs (directly actionable changes, identifying interaction patterns, looking beyond the reported); Reduce human error; Automate if possible	+
	Log Transfer	Remove unnecessary points of human intervention for mundane tasks (where possible)	−
	Planning approach	Supported incremental evolution of the interaction; Provides transparent justification for decisions	+
	Deploy early	The important issues are made clearer when observed *in situ*; Getting the system in front of the front-line workers leads to invaluable feedback; identifying both issues and ideas	+
	Interaction personalisation	Implement flexible components; Be open to opportunities for personalisation of the interaction, both in terms of the structure of the interaction, and the specific robot behaviours	+

The +/− column indicates whether our experience was positive or negative.

### 6.1 Team participation

One of the main findings of the initial consultation phase is that including all possible involved participants in the technology design process is crucial, including not only the workers but also patient partners who ensured that the robot is designed with families as a priority. It was also recognized that maintaining open communication and cooperation among the parties involved in the deployment is essential to ensure that the interaction is considered a viable option rather than an additional burden. In particular, the research assistants and HCPs, who are the front-line workers interacting with the SAR, were identified as critical participants in the process. They are the people who manage and operate the SAR on-site. They need to have high skills in handling the SAR, and their feedback regarding the practicality of the general implementation and the actual design of the user interface was essential. Furthermore, HCPs were also shown to play the role of advocates in the eyes of the caregivers and children, as parents’ trust in healthcare professionals and the clinical process could easily be extended to the SAR. In short, if the HCPs and the hospital deem the SAR useful and, more importantly, trustworthy, then parents also find it easy to trust.

This crucial role of HCPs and other medical personnel also carried over to the deployment process described here: ultimately, the system was only able to operate successfully in its final location thanks to the close interaction and collaboration between HCPs and the technical research team during the usability cycle phase.

### 6.2 Communication

Building and deploying a robot successfully requires effective communication between all of the involved teams. A key part of our approach was in defining a proper issue reporting and solving procedure, which included specific roles. For example, to handle the number of people involved, we identified a clinical research coordinator to manage the issues at each site. Reporting started when the RA who operated the robot filled out a form in which they identified key aspects of the interaction and reported any issues. Afterwards, the clinical research coordinator collected the form and any logs generated locally and shared them with the technical team. The research coordinator also discussed the findings with the technical team on a weekly basis. These meetings were effective at building a shared language and understanding between the teams. Every time a fix was needed, it was implemented, tested locally, and then transferred to the robots in the hospitals. The clinical research coordinator then determined whether re-training was required and monitored feedback. Overall, this process proved effective and placed the identification of priority items with the clinical team.

### 6.3 Environment

Another important lesson we learned was the challenge presented to the technical research team by their inability to have easy access to the deployment location, which meant that they relied even more on the interactions with the research assistants and HCPs, as well as their collaboration and feedback. Therefore, our extensive training sessions proved to be very helpful, not only for the final clinical trial phase (where, again, technical staff cannot be present) but also during the usability testing phase to ensure that the research assistants and HCPs both understood the technology and were confident in operating it in the field. The weekly meetings between the clinical research coordinators and the technical research teams were thus seen as very helpful.

### 6.4 System

The components of our system each generated a log each time the system was run. We designed an automated process for initially analysing and annotating the interaction logs. This tool processed the message level logs of the robot system and highlighted unexpected delays between events. It also reported sequences of events that were unexpected, such as missed messages. This greatly assisted in analysing the interactions. It was also straightforward for the operator to make a clear distinction between trial runs and study runs, which simplified the process of identifying logs. All of our logs were also automatically backed up in the robot system. This was particularly important in failed runs, where the access to partial runs was essential to determine the cause of failure.

One issue was that the logs required manual intervention to transfer them from the system at the medical site to the technical team. The intention was that the operator would save the log to the tablet. However, due to the lack of Internet access, the tablet had to be taken out of the ED to upload the files. Finding a way to enable this transfer to be processed automatically would have greatly streamlined the feedback process.

As described above, we used an AI planning-based approach to manage the interaction between the patient and the system. This approach supported incremental development of the scenario: as new interaction rules are added, the existing structure remains. This contrasts with traditional machine learning approaches, which would have required iterations of learning and appropriate training data at each stage. Furthermore, as the planning model is logic based, the justification for the selection of each behaviour can be extracted from the model, simplifying the process of debugging unexpected interactions.

Finally, the advantage of using an AI-based SAR that can respond more flexibly to the children was clearly demonstrated: feedback from caregivers and children showed that personalizing the robot’s content was very important, given that the need for the SAR’s interaction features and recommended behaviours varied greatly from one child to another. In addition, the needs and interaction expectations of the parents also varied greatly, which influenced the overall deployment strategy and required numerous adaptations to the robot system.

### 6.5 Design guidelines for deploying SAR in paediatric emergency departments

Based on the lessons learned from this robot’s deployment process, we propose the following guidelines for researchers and practitioners in this area.

1. Engage interested parties early and continuously. Involve clinical staff, patients, and caregivers from the outset to define the robot’s role, acceptable behaviours, and integration points in the care pathway.
2. Prioritise operator control. Provide intuitive, responsive controls that allow human operators to override automated decisions, particularly in emotionally sensitive or clinically urgent situations.
3. Design for environmental constraints. Account for noise, crowding, and interruptions typical of ED settings when specifying the input and output modalities for the system.
4. Plan for robustness and error handling. Implement strategies for managing incorrect or missing input information, including operator verification and conservative default behaviours.
5. Document and train for real-world use. Develop clear standard operating procedures and provide comprehensive operator training to ensure safe, consistent deployment.
6. Iterate with feedback loops Use structured feedback from each deployment cycle to refine the technical and interaction design elements.

## 7 Discussion

We have described the process of developing a socially assistive robot for use in the ED of two large tertiary care children’s hospitals, with the goal of supporting HCPs while children are undergoing intravenous insertion. The details of the robot system were developed and refined in a thorough co-design process involving technical team members, clinical staff, HCPs, children, and parents and caregivers from the two clinical sites, and an initial version of the system was developed. Transferring the robot from this original lab-based version into a system which was able to be used by research assistants independently and reliably took a significant amount of time and effort and required extensive and intensive collaboration between the clinical and technical research teams. In the end, the robot was deemed to be ready for its final deployment, and a randomized clinical trial has been carried out at both clinical sites comparing observed and reported pain and distress between children who interact with the robot and those who receive the standard care from each site, while also gathering other qualitative and quantitative measures to assess the impact on parents, caregivers, and HCPs. The analysis of the clinical trial data is ongoing at the time of writing this paper; details are available at (ClinicalTrials.gov, 2024).

The extended length of the deployment and usability testing process has helped to gather rich feedback and insights during the two cycles of usability testing, and a number of lessons have been learned, from which we have derived guidelines which can inform future robot deployments in other clinical workplaces and similar contexts. A number of problems arose and a significant amount of feedback was given after the first usability cycle from HCPs, children, and their caregivers. Instead of quickly and reactively trying to update the system, we were able to take time with the implementation to ensure that decisions were well-motivated and that all participants were able to contribute. Given the well-established synergy and the tools deployed in place, the entire research team was able to achieve overall positive feedback after the second round of usability testing.

A key contribution of this work lies in its comprehensive account of the pathway from initial concept to real-world deployment, including iterative co-design with all interested parties, technical integration, usability testing, and integration into an active clinical environment. In the SAR literature, many papers report only final outcomes, leaving the intermediate steps under-documented. By making these steps explicit, and by proposing concrete guidelines arising from the lessons learned from the entire process, we provide a transferrable framework that other research groups can adapt to their own settings, regardless of the specific deployment context. This process-level contribution stands independently of the clinical trial, which will be reported in full detail in a separate publication.

## 8 Future work

We have focused on the design, implementation, and deployment of a single socially assistive robot, carrying out specific tasks during IV insertion in two identified paediatric emergency departments. While we have drawn several general conclusions from the experience, including concrete design guidelines for working in this space, we can also identify several avenues for future exploration. On the clinical side, we could extend the robot to other clinical contexts, such as surgical recovery units or oncology wards, to assess the generalisability of the system and the defined behaviours, following the above guidelines while developing the details of the revised system. On the technical side, improved sensing technology—such as thermal imaging or physiological sensors—could enhance the accuracy of the robot’s affective state estimation, in turn opening up the possibility of further automating the behaviour selection process, reducing operator workload while maintaining safety and appropriateness. Finally, additional longitudinal studies, including and building on the current clinical trial, will be essential to evaluate the sustained impact of the system on patient outcomes and staff workflows.

## Data Availability

The original contributions presented in the study are included in the article/supplementary material, further inquiries can be directed to the corresponding author.

## Footnotes

1 Full technical details can be found in (Foster et al., 2023; Ramirez Duque et al., 2024; Lindsay et al., 2024a; Lindsay et al., 2024b).
